# *Porphyromonas gingivalis* hijacks mitophagy and lysosomal function to persist in endothelial cells

**DOI:** 10.3389/fcimb.2025.1613366

**Published:** 2025-08-08

**Authors:** Cheng Zheng, Jianmin Huang, Shengming Xu, Bin Lu, Hanxin Que, Tianhao Chen, Yubo Hou, Linlin He, Xia Fan, Fa-Ming Chen, Yi Wang, Hui Deng

**Affiliations:** ^1^ Institute of Stomatology, School and Hospital of Stomatology, Wenzhou Medical University, Wenzhou, Zhejiang, China; ^2^ Department of Periodontology, School and Hospital of Stomatology, Wenzhou Medical University, Wenzhou, Zhejiang, China; ^3^ State Key Laboratory of Oral & Maxillofacial Reconstruction and Regeneration, National Clinical Research Center for Oral Diseases, Shanxi International Joint Research Center for Oral Diseases, Department of Periodontology, School of Stomatology, The Fourth Military Medical University, Xi’ an, Shanxi, China; ^4^ Department of Orthodontics, School and Hospital of Stomatology, Wenzhou Medical University, Wenzhou, Zhejiang, China

**Keywords:** *Porphyromonas gingivalis*, endothelial cells, mitophagy, lysosomal function, xenophagy

## Abstract

**Introduction:**

The direct infection of endothelial cells by *Porphyromonas gingivalis* (*P. gingivalis*), a keystone periodontal pathogen, has been implicated in the development of atherosclerosis. While non-selective autophagy facilitates its intracellular persistence in endothelial cells, the role of selective autophagy in this process remains unclear. This study investigated whether *P. gingivalis* hijacks mitophagy and lysosomes to persist in endothelial cells.

**Methods:**

Human aortic endothelial cells (HAECs) were infected with *P. gingivalis* for 24 h. Mitophagy was detected by Western Blotting (WB), immunofluorescence, and transmission electron microscopy. Lysosomal function was assessed by acridine orange staining, lysosensor staining, and WB. The effects of mitophagy and lysosomes on *P. gingivalis* intracellular survival were evaluated by antibiotic protection assays and SYTO-9 staining.

**Results:**

Our data demonstrated that *P. gingivalis* initiates PTEN­induced putative kinase 1 (PINK1)-Parkin-mediated mitophagy in HAECs, leading to increased formation of autophagosomes and mitophagosomes, but disrupted autophagy/mitophagy flux. This blockage of autophagy/mitophagy flux was linked to lysosomal dysfunction, characterized by increased lysosome number, lysosomal membrane permeabilization, disruption of the lysosomal acidic environment, and decreased enzymatic activity. Additionally, antibiotic protection assays and SYTO-9 staining further revealed that *P. gingivalis* promotes its intracellular survival in endothelial cells by initiating mitophagy and impairing lysosomal function. Furthermore, the mitophagy activator decreased the co-localization of *P. gingivalis* with microtubule-associated protein 1 light chain 3 (LC3)-p62, LC3-NDP52, and lysosomal-associated membrane protein 1 (LAMP1), suggesting that *P. gingivalis*-initiated mitophagy inhibited xenophagosome formation and autophagosome/xenophagosome-lysosome fusion.

**Conclusion:**

Our findings reveal that *P. gingivalis* may promote its intracellular survival in endothelial cells by initiating PINK1-Parkin-mediated mitophagy and impairing lysosomal function, thereby suppressing xenophagosome formation and xenophagic degradation. This study provides new insights into the mechanisms by which *P. gingivalis* persists in endothelial cells and its potential role in atherosclerosis progression.

## 1 Introduction

*Porphyromonas gingivalis* (*P. gingivalis*), a keystone pathogen in periodontal disease, is recognized for its ability to enter the bloodstream and colonize extra-oral tissues (Forner et al., 2006). Notably, the DNA of *P. gingivalis* has been identified in human atherosclerotic plaques, providing direct evidence of its association with atherosclerosis (Haraszthy et al., 2000). Animal studies further support this link, showing that *P. gingivalis* infection accelerates the progression of atherosclerotic lesions (Li et al., 2002; Brodala et al., 2005). Endothelial cells, which play a crucial role in atherosclerosis, are directly targeted by *P. gingivalis* (Gimbrone and García-Cardeña, 2016). This bacterium adheres to and invades endothelial cells, evades immune surveillance, and persists in a dormant state or replicating state, contributing to the pathology of atherosclerosis (Zheng et al., 2021). However, the mechanisms by which *P. gingivalis* survives and evades immune surveillance within endothelial cells remain largely unexplored.

Autophagy is the process by which cells eliminate intracellular pathogens, damaged organelles, and misfolded proteins. It begins when a phagophore, encapsulates cytoplasmic components to form autophagosomes. The autophagosomes then fuse with lysosomes to become autolysosomes, where their contents are degraded by lysosomal enzymes (Mizushima and Komatsu, 2011; Dikic and Elazar, 2018). Autophagy can be categorized into non-selective or selective. In non-selective autophagy, cells indiscriminately engulf and degrade parts of cytosolic components, such as intracellular bacteria and host cell proteins. Interestingly, upon invading endothelial cells and gingival epithelial cells, *P. gingivalis* triggers a non-selective autophagy pathway and directs itself into rich-nutrient autophagosomes to evade immune surveillance and promote its survival (Bélanger et al., 2006; Lee et al., 2018).

Selective autophagy targets specific components, such as damaged mitochondria (mitophagy) and intracellular bacteria (xenophagy), for degradation (Wang et al., 2023). Mitophagy is primarily regulated through a ubiquitin-dependent signaling pathway involving two major proteins: PTEN­induced putative kinase 1 (PINK1) and E3 ubiquitin ligases Parkin. PINK1-Parkin-mediated mitophagy relies on ubiquitin-binding adaptor proteins such as p62, NDP52, and optineurin (Lazarou et al., 2015). Interestingly, mitophagy shares several molecular components, including those adaptor proteins, with antimicrobial selective autophagy (xenophagy), and they may compete for the same molecules (Stolz et al., 2014; Song et al., 2022; Rubio-Tomás et al., 2023).

In addition to the non-selective autophagy, certain bacteria have evolved mechanisms to exploit selective autophagy to support their survival. For instance, *Burkholderia pseudomallei*, *Neisseria gonorrhoeae*, and *Listeria monocytogenes* manipulate mitophagy to limit mitochondrial reactive oxygen species (mtROS) production, helping them evade host immune defenses (Zhang et al., 2019; Gao et al., 2024; Nan et al., 2024). Additionally, *P. gingivalis* has been shown to cleave NDP52 through its gingipains, enabling the bacterium to resist xenophagic degradation (Shiheido-Watanabe et al., 2023). Similarly, *Mycobacterium bovis* (*M. bovis*) inhibits host xenophagy by inducing mitophagy, thus enhancing its intracellular survival (Song et al., 2022).

Being the organelle responsible for the final degradation stage of autophagy, the lysosome is also vital in determining the fate of pathogens (Ballabio and Bonifacino, 2020). Some bacteria, such as *Escherichia coli* and *Helicobacter pylori*, survive within autophagosomes by reducing hydrolase activity through increasing lysosomal pH (Miao et al., 2015; Naskar et al., 2023). Recent studies suggested that *P. gingivalis* promotes lysosomal efflux in gingival epithelial cells, thereby preventing autophagosome-lysosome fusion and allowing the bacteria to survive (Liu et al., 2023). However, it remains uncertain whether *P. gingivalis* exploits mitophagy and lysosomal function to promote survival within endothelial cells.

This study aims to explore how *P. gingivalis* promotes its intracellular survival in endothelial cells by investigating the roles of mitophagy and lysosomal function. The findings of our study could provide new insights into the intracellular survival strategies of *P. gingivalis*.

## 2 Materials and methods

### 2.1 Reagents and primary antibodies

The reagents used in this study include carbonyl cyanide m-chlorophenylhydrazone (CCCP, MCE, Cat# HY-10094), ML-SA1 (MCE, Cat# HY-108462), Bafilomycin A1 (BafA1, MCE, Cat# HY-100558), and mitochondrial division inhibitor 1 (Mdivi-1, MCE, Cat# HY-15886). Other reagents included SYTO-9 (Invitrogen, Thermo Fisher Scientific, Cat# S34854), Actin-Tracker Red-Rhodamine (Beyotime Biotechnology, Cat# C2207S), acridine orange hydrochloride (AO, MCE, Cat# HY-101879), MitoSOX (Invitrogen, Thermo Fisher Scientific, Cat# M36007), and Mito-Tracker Red CMXRos (Beyotime Biotechnology, Cat# C1035). The details of primary antibodies for Western Blotting and immunofluorescence are described in **Supplementary Tables 1**, **2** respectively.

### 2.2 Cell culture

HAECs (iCell Bioscience, Cat# C1252, China) were cultured in endothelial cell medium (ECM) supplemented with 5% fetal bovine serum and 1% endothelial cell growth supplement. The cells were maintained in a humidified incubator at 37°C with 5% CO_2_.

### 2.3 Bacteria culture and fluorescent labeling

*P. gingivalis* W83 (ATCC, Manassas, VA, USA) was cultured on anaerobic blood agar plates within a chamber with 85% N2, 5% H_2_, and 10% CO_2_ at 37°C for 5–7 days. The bacteria were then inoculated into a liquid broth of brain heart infusion (BHI) supplemented with hemin (5 μg/mL) and vitamin K (5 μg/mL) and grown for over 24 h at 37°C. *P. gingivalis* was labeled with SYTO-9 according to the protocol provided by the manufacturer. HAECs were seeded onto coverslips in 24-well plates and co-cultured with SYTO-9-labeled *P. gingivalis* at a multiplicity of infection (MOI) of 1:100 for 2 h or 24 h. After incubation, the cells were stained with Actin-Tracker and DAPI for further analysis.

### 2.4 Immunofluorescence and imaging

HAECs were seeded onto coverslips in 24-well culture plates and pretreated with dimethyl sulfoxide (DMSO) (0.25%), BafA1 (20 nM), Mdivi-1 (25 μM), or CCCP (20 μM) for 2 h, or treated with ML-SA1 (10 μM) during *P. gingivalis* infection. Following pretreatments, cells were infected with *P. gingivalis* (MOI=100) for 24 h. Cells were sequentially fixed, permeabilized, and blocked to prepare samples for immunofluorescence. They were then incubated with the primary antibodies (**Supplementary Table**), followed by incubation with the secondary antibody. Mito-Tracker Red CMXRos was applied to stain mitochondria before fixation. Images were captured using a Leica Stellaris 5 confocal laser scanning microscope (CLSM, Leica Microsystems, Waltham, MA, USA) with a 63X oil immersion lens. Approximately 10–15 random images were acquired for each treatment group. The experiment was conducted in triplicate.

### 2.5 Antibiotic protection assays

HAECs were seeded at a density of 3 × 10^5^ on 6-well plates. Cells were pretreated with DMSO (0.25%), BafA1 (20 nM), Mdivi-1 (25 μM), or CCCP (20 μM) for 2 h, or treated with ML-SA1 (10 μM) during *P. gingivalis* infection. Following pretreatments, cells were infected with *P. gingivalis* (MOI=100) for 2 h or 24 h. At each collection time point, HAECs were washed and then incubated in ECM containing gentamicin (300 μg/mL) and metronidazole (200 μg/mL) for 1 h to eliminate extracellular bacteria. The cells were subsequently lysed with iced double-distilled H_2_O (ddH_2_O). Lysates were serially diluted in ddH_2_O and plated on blood agar plates. Colony-forming units (CFUs) were determined after incubation to assess the number of intracellular bacteria. The experiment was conducted in triplicate.

### 2.6 Western blotting

Total protein extracts were prepared from cells using a modified RIPA buffer, containing freshly added protease and phosphatase inhibitors. Following the instructions provided by the manufacturer, protein concentration was determined using a BCA assay kit (Beyotime Biotechnology, Shanghai, China). The extracted proteins were separated by sodium dodecyl sulfate-polyacrylamide gel electrophoresis (SDS-PAGE). Polyvinylidene fluoride (PVDF) membranes with transferred proteins were blocked in 5% non-fat dry milk and then incubated with the primary antibodies (**Supplementary Table**), followed by secondary antibody incubation. Protein expression levels were normalized to GAPDH and are expressed as fold changes relative to the control. The experiment was conducted in triplicate.

### 2.7 Transmission electron microscopy observation

Cells were fixed overnight at 4°C using 2.5% glutaraldehyde. After fixation, the cells were washed with sodium cacodylate buffer and fixed in 1% osmium tetroxide for 1 h, followed by dehydration with acetone. The cell pellet was infiltrated with resin and polymerized overnight at 60°C. Resin-embedded samples were sectioned into 70-nm ultrathin slices, and images were captured using a FEI Tecnai G2 F20 electron microscope (Thermo Fisher Scientific, USA). Each experiment was performed in triplicate.

### 2.8 LysoTracker green staining

LysoTracker Green staining (Beyotime Biotechnology, Shanghai, China) was used to label and quantify the lysosomes. After *P. gingivalis* treatment, HAECs were stained with LysoTracker Green (75 nM) at 37°C for 30 min, and then observed and imaged by CLSM.

### 2.9 siRNA transfection

Human *Pink1*-targeting siRNA plasmids were from TsingKe Biotech (China). The primers of each siRNA plasmid are as follows: si*Pink1–*1 with the forward primer 5’ -GAGACCUGAAAUCCGACAA- 3’ and the reverse primer 5’-UUGUCGGAUUUCAGGUCUC-3’; si*Pink1–*2 with the forward primer 5’ -GCCAUCUUGAACACAAUGA- 3’ and the reverse primer 5’-UCAUUGUGUUCAAGAUGGC-3’; si*Pink1–*3 with the forward primer 5’ -GCAAAUGUGCUUCAUCUAA- 3’ and the reverse primer 5’-UUAGAUGAAGCACAUUUGC-3’.

*Pink1* siRNA and negative control siRNA plasmids were transfected into HAECs at 20nM using Lipofectamine 3000 (Thermo Fisher Scientific, USA) following the manufacturer’s instructions. The transfection medium was replaced with fresh complete medium after 4 h, and the cells were transfected for 48 h. The efficiency of PINK1 silencing was detected by Western blot.

### 2.10 Acid phosphatase activity

After 24 h of exposure to *P. gingivalis* (MOI=100), the intracellular fluoride-resistant acid phosphatase activity was measured using a commercially available kit, according to the instructions provided by the manufacturer (ACP activity assay kit, Beyotime Biotechnology, Shanghai, China).

### 2.11 Lysosomal membrane permeability

Acridine orange (AO) staining was utilized to evaluate lysosomal membrane permeability (LMP). In brief, HAECs were exposed to *P. gingivalis* (MOI=100) for 24 h. As a positive control, cells pretreated with BafA1 (20 nM for 2 h) were included. Following this, the cells were stained with AO solution (5 μg/mL) for 20 min at 37°C and then observed under a fluorescence microscope.

### 2.12 Measurement of mtROS

HAECs were pretreated with DMSO (0.25%), Mdivi-1 (25 μM), or CCCP (20 μM) for 2 h. Following pretreatments, cells were infected with *P. gingivalis* (MOI=100) for 24 h. Intracellular mtROS level was detected using MitoSOX (5 μM) for 15–30 min at 37°C and then observed under a fluorescence microscope. Each experiment was performed in triplicate.

### 2.13 Statistical analysis

The normal distribution of data was checked by the Shapiro–Wilk test, and all data sets in the study passed the normality distribution test. Statistical significance was determined by using an Unpaired t-test (two-tailed). Comparisons among multiple groups were demonstrated utilizing one-way ANOVA along with Tukey’s *post hoc* test. Results were presented as mean ± SEM from a minimum of three biological replicates. *P*<0.05 was considered statistically significant, and all statistical analyses were performed with GraphPad Prism 9.0 (GraphPad Software, Boston, MA, USA).

## 3 Results

### 3.1 *P. gingivalis* initiated mitophagy and increased accumulation of mitophagosomes in endothelial cells

**Figure 1:**
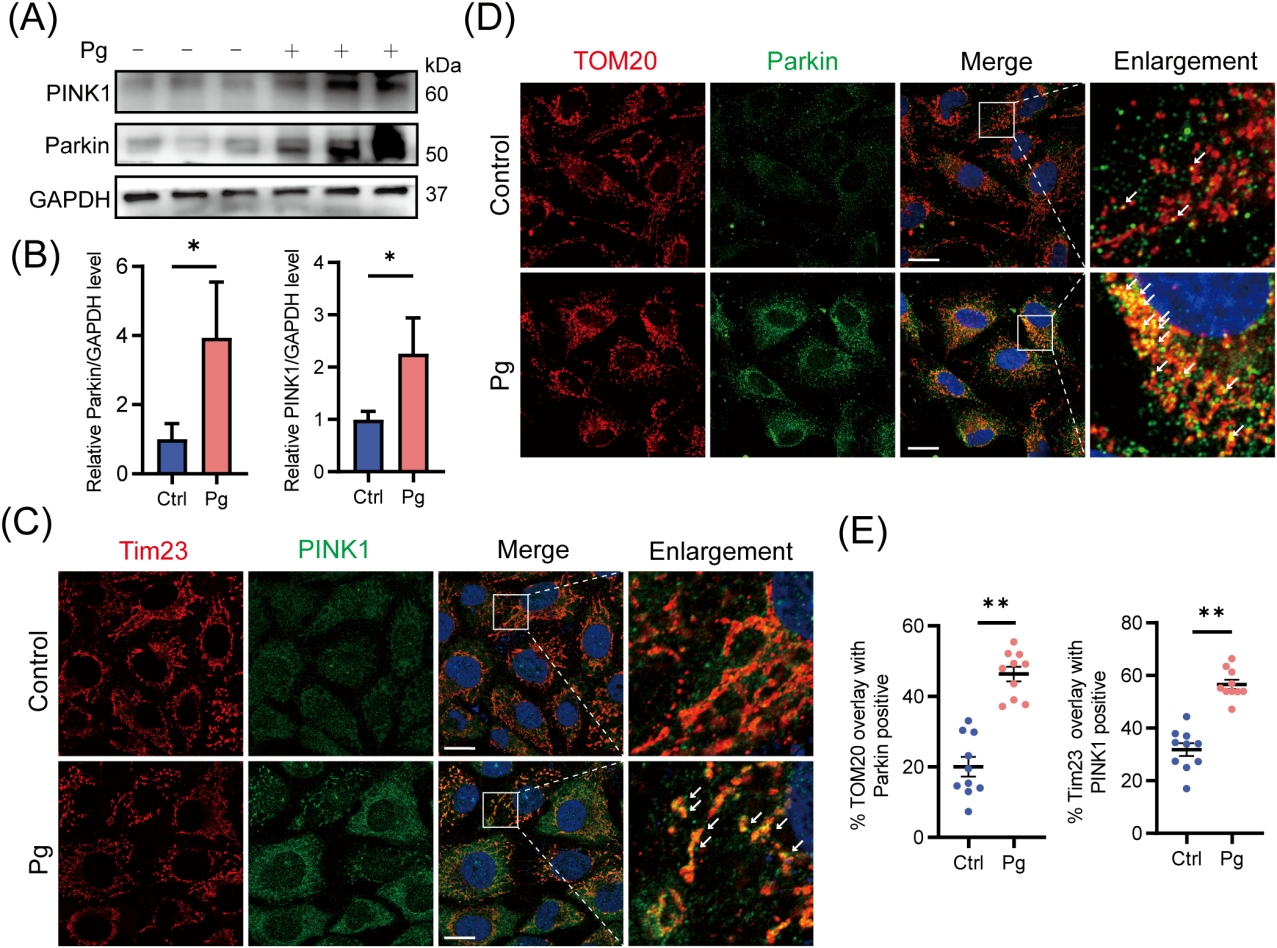
Initiation of PINK1-Parkin-mediated mitophagy upon *Porphyromonas gingivalis* stimulation at an MOI of 100 for 24 h in HAECs. **(A)** Representative western blot and **(B)** quantification of Parkin and PINK1 protein expression in control or Pg-infected HAECs (n=3 per group). **(C, D)** Representative images and **(E)** quantification of Tim23 (red) -PINK1 (green) and TOM20 (red) -Parkin (green) colocalization by confocal laser scanning microscope (CLSM). Scale bar: 15 μm. (n = 10–15 cells per group). Data are presented as the mean ± SEM. *P < 0.05 and **P< 0.01, compared with the control.

Following exposure to *P. gingivalis* (MOI=100) for 24 h in HAECs, we observed upregulation of proteins PINK1 and Parkin (**Figures 1A, B**), along with enhanced immunofluorescence co-localization of these proteins with mitochondria (**Figures 1C–E**), suggesting the activation of mitophagy-related signaling molecules. To validate these results, we assessed mitochondrial membrane potential (MMP) and mtDNA copy number. Our results showed a dose- and time-dependent decline in MMP (**Supplementary Figures 1A–D**) and a reduction in mtDNA copy number (**Supplementary Figure 1E**), suggesting that *P. gingivalis* induces mitochondrial dysfunction. These findings showed that *P. gingivalis* impaired mitochondrial function and initiated PINK1-Parkin-mediated mitophagy in endothelial cells.

**Figure 2:**
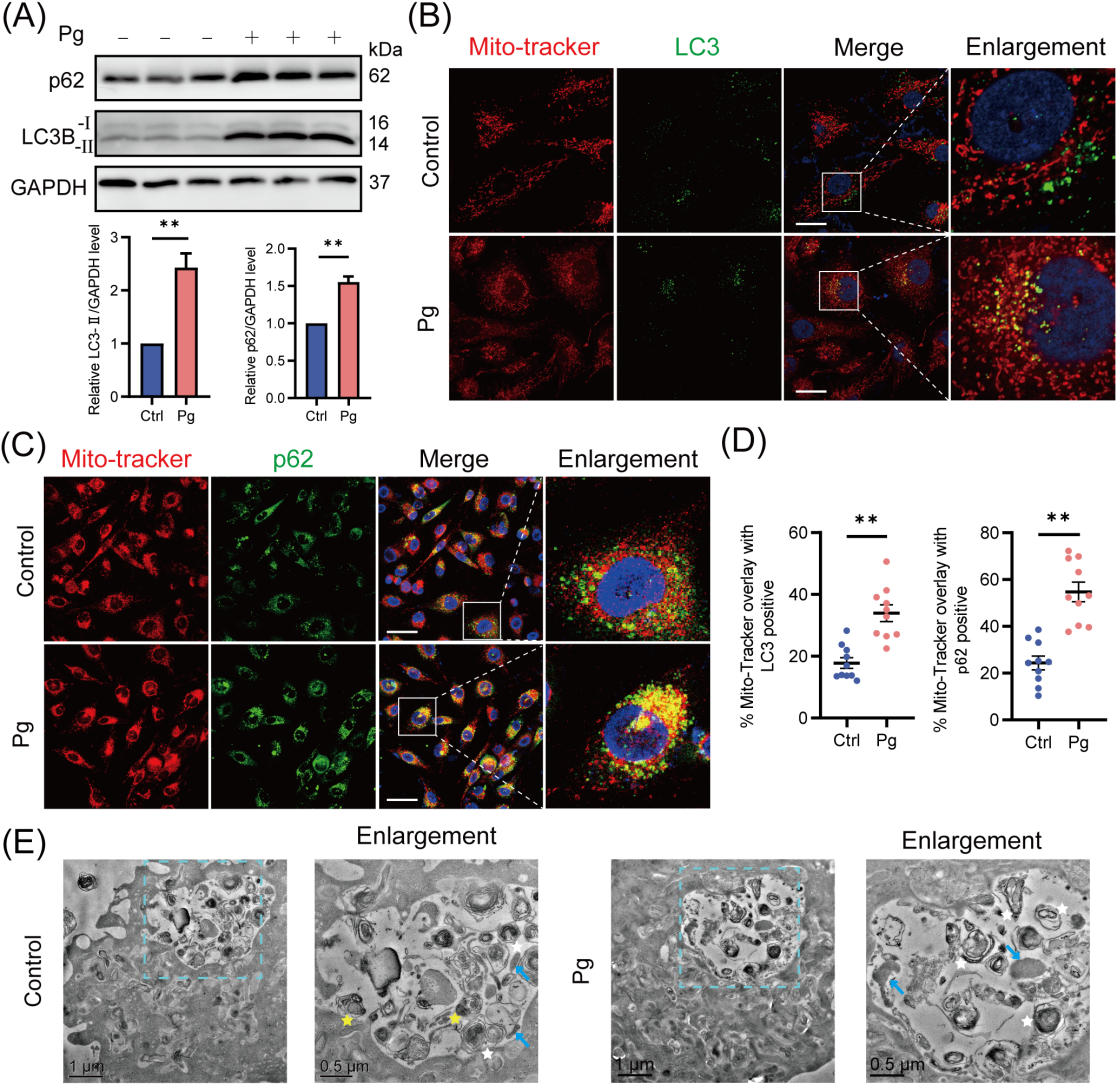
Mitophagosome formation accumulated upon *Porphyromonas gingivalis* infection. HAECs were infected with Pg at an MOI of 100 for 24 h. **(A)** Representative western blot and quantification of LC3-I/II and p62 protein expression in control or Pg-infected HAECs (n = 3 per group). **(B)** Representative images of Mito-Tracker (red) and LC3 (green) colocalization by CLSM. Scale bar: 15 μm. **(C)** Representative images of Mito-Tracker (red) and p62 (green) colocalization by CLSM. Scale bar: 30 μm. **(D)** The colocalization of MitoTracker-LC3 and MitoTracker-p62 was quantified (n = 10–15 cells per group). **(E)** Transmission electron microscopy (TEM) images revealed the mitophagosome-like vacuoles and mitophagolysosome-like vacuoles in control or Pg-infected HAECs (blue arrow: mitochondria, white star: mitophagosome, yellow star: mitophagolysosome, containing autophagosome). Data are presented as the mean ± SEM. **P< 0.01, compared with the control.

Mitophagy involves the formation of mitophagosomes, which are subsequently fused with lysosomes and degraded (Pickles et al., 2018). To investigate the development of autophagy/mitophagy, we examined the expression of lipidated microtubule-associated protein 1 light chain 3 proteins (LC3-II, autophagy formation marker) and p62 (autophagy degradation marker) (Klionsky et al., 2021) in HAECs exposed to *P. gingivalis* for 24 h, finding that *P. gingivalis* significantly enhanced the expression levels of LC3-II and p62 protein (**Figure 2A**). Given that p62 levels negatively correlate with autophagic activity (Klionsky et al., 2021), these results suggested that *P. gingivalis* may trigger the autophagy/mitophagy process but block lysosomal degradation.

Furthermore, mitochondria co-localized with LC3 or cargo protein p62/NDP52 were analyzed to confirm the mitophagosome formation in HAECs. Upon infection with *P. gingivalis*, we observed an increase in the recruitment of p62/NDP52 to mitochondria and a significant rise in the percentage of mitophagosomes (yellow puncta/red puncta%) (**Figures 2B–D**; **Supplementary Figures 2A, B**), indicating enhanced formation of mitophagosomes. Next, transmission electron microscopy (TEM) was used to check subcellular structures within HAECs. We observed an increase in mitophagosome-like vacuoles with characteristic double-membrane structures, while mitophagolysosome-like vacuoles were rare in the *P. gingivalis*-treated group (**Figure 2E**). In summary, these results confirmed that *P. gingivalis* promotes an unhealthy accumulation of mitophagosomes in HAECs.

### 3.2 *P. gingivalis* increased the accumulation of mitophagosomes by reducing mitophagy/autophagy flux

**Figure 3:**
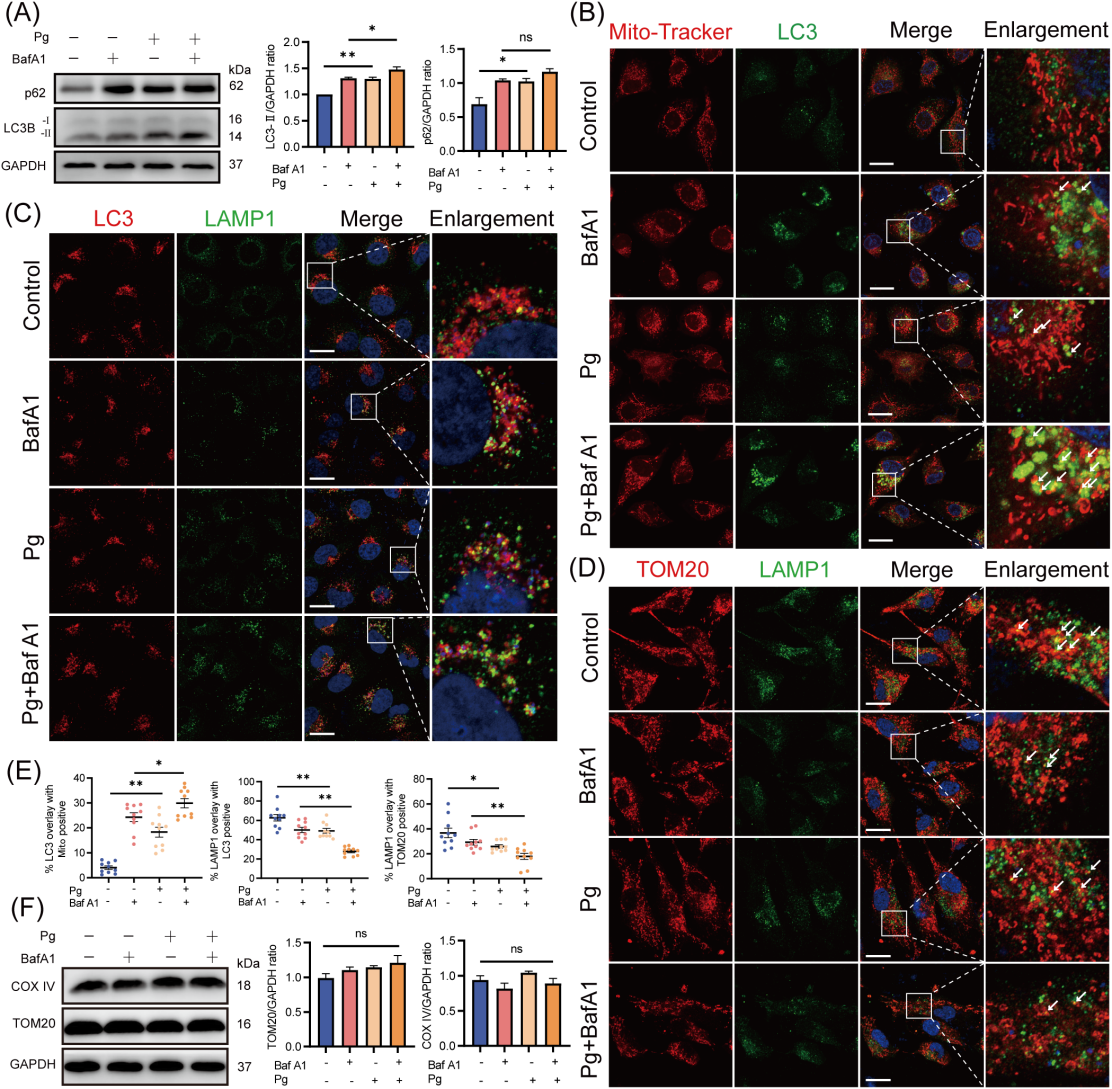
*Porphyromonas gingivalis* increased the accumulation of mitophagosomes by reducing mitophagy/autophagy flux. HAECs were pretreated with or without BafA1 (autophagosome-lysosome fusion inhibitor) for 2 h before Pg exposure (MOI=100, 24 h). **(A)** Representative western blot and quantification of LC3-I/II and p62 protein expression (n = 3 per group). **(B-D)** Representative images of Mito-Tracker (red)- LC3 (green), LC3 (red)- LAMP1 (green), and TOM20 (red)- LAMP1 (green) colocalization by CLSM. Scale bar: 15 μm. **(E)** The colocalization of MitoTracker-LC3, LC3-LAMP1, and TOM20-LAMP1 was quantified (n = 10–15 cells per group). **(F)** Representative western blot and quantification of mitochondrial marker COX IV and TOM20 protein expression (n = 3 per group). Data are presented as the mean ± SEM. *P < 0.05, and **P< 0.01, ns, not significant.

To determine whether the accumulation of mitophagosomes is due to increased mitophagosome formation or impaired lysosomal clearance, HAECs were pretreated with the autophagosome-lysosome fusion inhibitor BafA1 for 2 h. After BafA1 treatment, we observed an increased LC3-II protein expression level and enhanced co-localization of LC3 with mitochondria in *P. gingivalis*-infected group compared with the uninfected ones (**Figures 3A, B, E**), suggesting that *P. gingivalis* promotes mitophagosome formation. Interestingly, the total accumulation of mitophagosomes (a comparison of LC3-II expression levels in control and *P. gingivalis* treatment groups) exceeded the level of mitophagosome formation, implying that *P. gingivalis* inhibited mitophagosome degradation in endothelial cells. Supporting the above observation on LC3 levels, there was no significant change in p62 protein levels between BafA1-treated and untreated HAECs exposed to *P. gingivalis* (**Figure 3A**), suggesting mitophagy/autophagy flux was reduced.

Additionally, we observed reduced co-localization of LC3 with lysosomal-associated membrane protein 1 (LAMP1, lysosomal marker) and translocase of outer mitochondrial membrane 20 (TOM20) with LAMP1 in *P. gingivalis*-infected HAECs (**Figures 3C–E**), suggesting that *P. gingivalis* inhibited mitophagosome-lysosome fusion. The expression levels of mitochondrial proteins TOM20 and COX IV did not decrease after *P. gingivalis* exposure (**Figure 3F**), indicating that damaged mitochondria were not effectively degraded. Overall, these results demonstrated that *P. gingivalis* increased the formation of mitophagosomes and reduced mitophagy/autophagy flux.

### 3.3 *P. gingivalis* reduced mitophagy/autophagy flux by impairing lysosomal function

**Figure 4:**
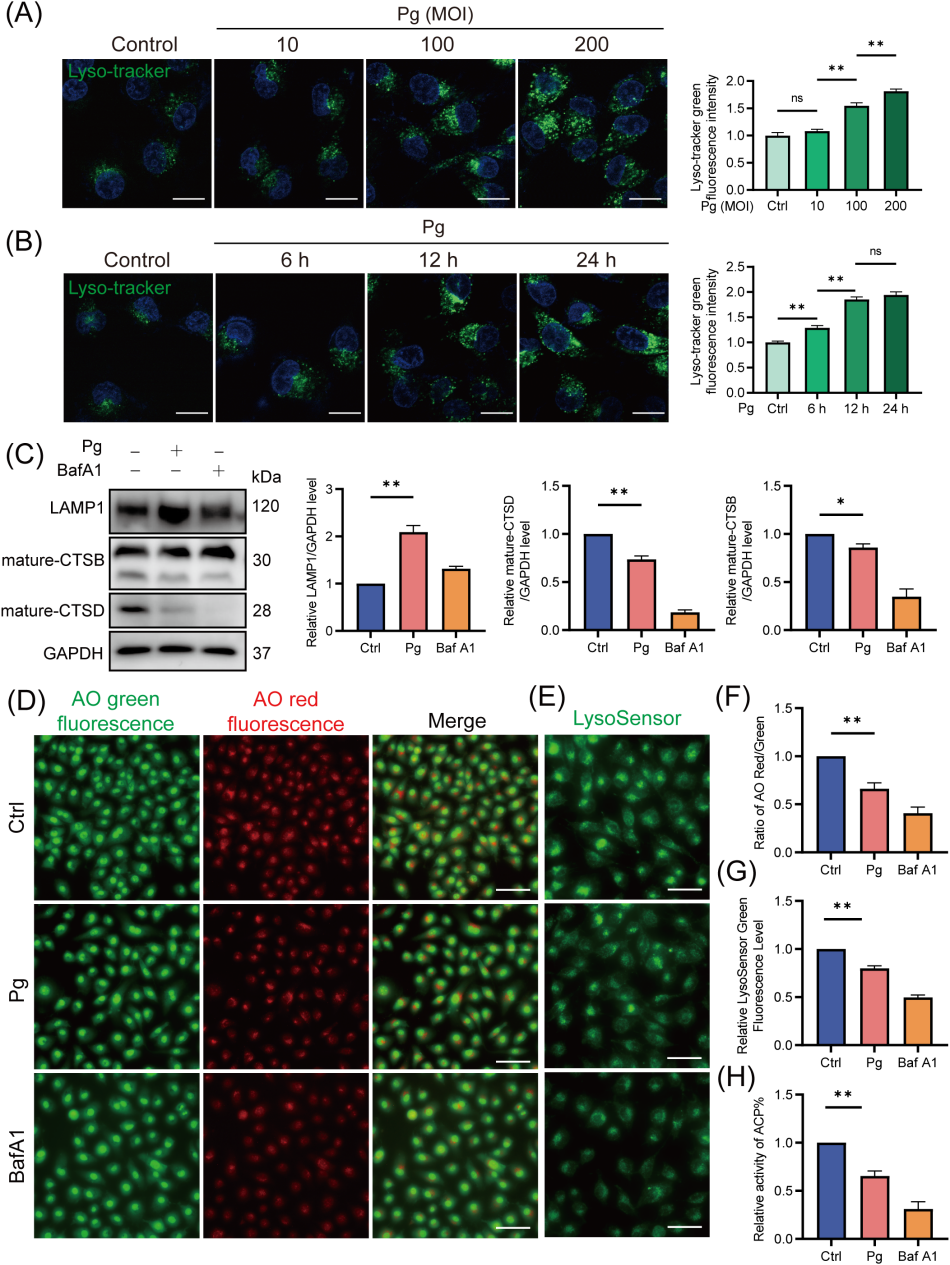
*Porphyromonas gingivalis* impaired lysosomal function in endothelial cells. HAECs were treated with Pg (MOI=100, 24 h) or not, and were pretreated with BafA1 for 2 h as a positive control. **(A, B)** Representative fluorescence images of Lyso-Tracker Green staining and the corresponding semi-quantification showed a dose- and time-dependent increase in lysosomal quantity in Pg-infected HAECs. Scale bar: 15 μm. **(C)** Representative western blot and quantification of mature-CTSB, mature-CTSD, and LAMP1 protein expression (n = 3 per group). **(D)** Representative fluorescence images of AO staining indicated the increase of LMP upon Pg stimulation. Scale bar: 200 μm. **(E)** Representative fluorescence images of LysoSensor green staining reflected changes in lysosomal pH value. Scale bar: 100 μm. **(F)** The relative LysoSensor green fluorescence intensity. **(G)** Ratio of AO red/green fluorescence intensity. **(H)** Acid phosphatase activity detection showed the activity of ACP. Data are represented from at least three independent experiments (mean ± SEM). *P < 0.05, and **P< 0.01, ns, not significant.

A healthy lysosome is essential for the final degradation of autophagic substrates (Settembre et al., 2013). Therefore, we assessed the impact of *P. gingivalis* on lysosomal function in endothelial cells. Our results demonstrated a time- and dose-dependent increase in LysoTracker fluorescence intensity (**Figures 4A, B**) and LAMP1 protein expression (**Supplementary Figures 3A, B**) in the *P. gingivalis* treatment group, suggesting an elevation in lysosomal quantity due to *P. gingivalis* infection. These findings suggested that *P. gingivalis* is unlikely to reduce autophagy/mitophagy flux by promoting lysosomal efflux. Moreover, *P. gingivalis* exposure led to a decrease in the AO red/green ratio (**Figures 4D, F**), indicating increased lysosomal membrane permeability (LMP), which suggested that *P. gingivalis* may compromise lysosomal integrity. Additionally, the reduced LysoSensor fluorescence intensity suggested an elevated lysosomal pH value (**Figures 4E, G**). Given the observed lysosomal alkalization induced by *P. gingivalis*, it is necessary to further examine whether lysosomal hydrolases were affected, as hydrolase activity is responsible for waste disposal in an acidic environment. Our results revealed a reduction in the expression of mature cathepsin B (CTSB) and cathepsin D (CTSD) (**Figure 4C**), along with decreased acid phosphatase (ACP) activity (**Figure 4H**). Taking together, these findings indicated that *P. gingivalis* induces lysosomal dysfunction in endothelial cells, as evidenced by increased LMP, disruption of lysosomal acidity, and impaired degradative capacity.

### 3.4 *P. gingivalis* initiated mitophagy and impaired lysosomal function to promote its intracellular survival

**Figure 5:**
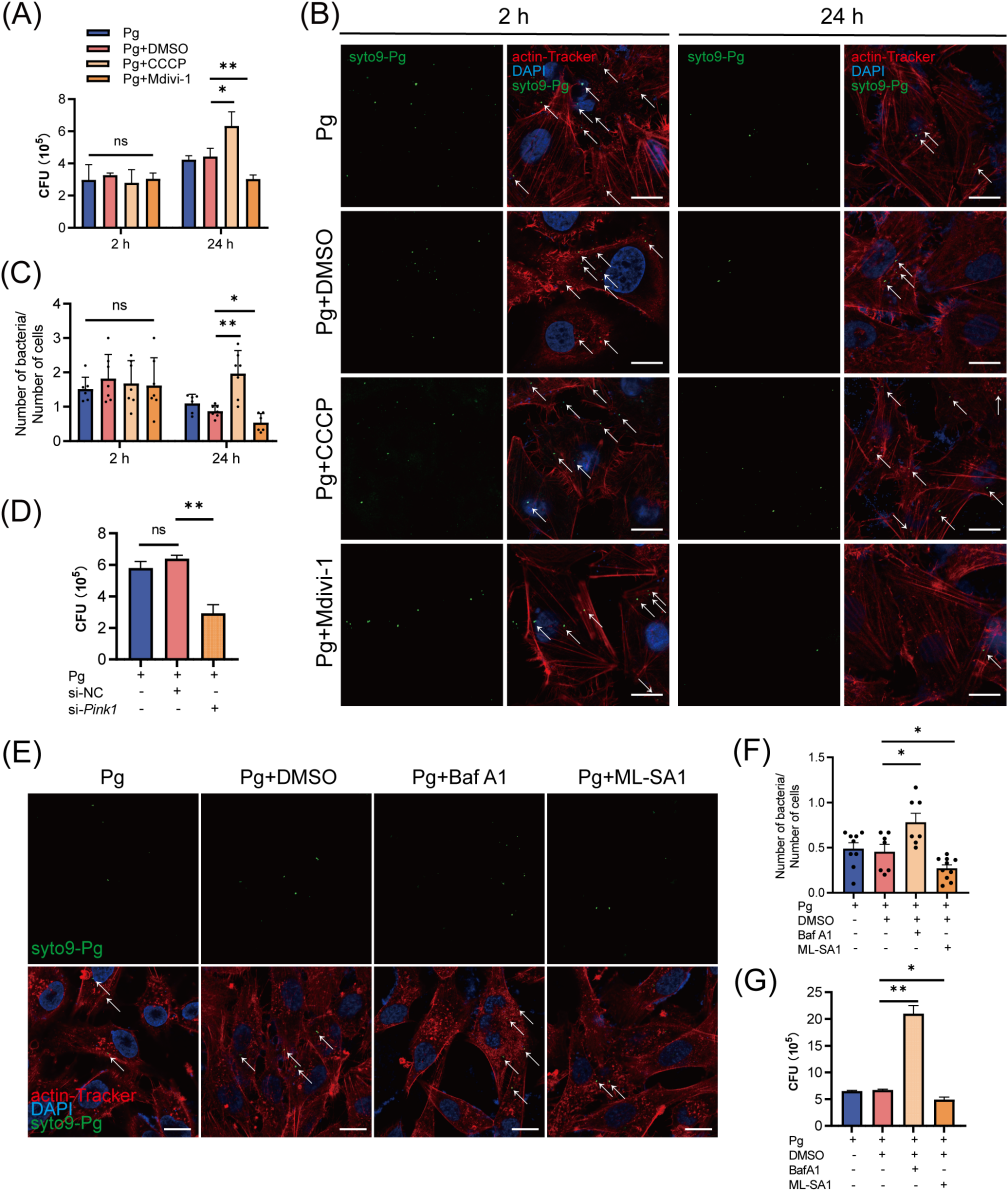
The initiation of mitophagy and lysosomal dysfunction increased the intracellular survival of *Porphyromonas gingivalis* in endothelial cells. **(A)** HAECs were treated with DMSO, CCCP (activation of mitophagy), or Mdivi-1 (inhibition of mitophagy), then infected with Pg (MOI = 100) for 2 h or 24 h. Pg intracellular survival was measured by antibiotic protection assays. **(B)** Representative fluorescence images of intracellular SYTO9-stained Pg load by CLSM. Scale bar: 15 μm. **(C)** The ratio of SYTO9-stained Pg to cell number. Cells in at least 6 fields were counted in each group. **(D)** HAECs were transfected with *Pink1* siRNA (20 nM) or negative control siRNA (20 nM) for 48 h and then infected with Pg (MOI = 100). Intracellular Pg load was measured by antibiotic protection assays. **(E)** HAECs were treated with DMSO, BafA1 (inhibition of lysosomal function), or ML-SA1 (activation of lysosomal function), then infected with Pg (MOI = 100) for 24 h. Representative fluorescence images of intracellular SYTO9-stained Pg load by CLSM. Scale bar: 15 μm. **(F)** The ratio of SYTO9-stained Pg to cell number. Cells in at least 6 fields were counted in each group. **(G)** Pg intracellular survival was measured by antibiotic protection assays. Data are presented as the mean ± SEM. *P < 0.05, and **P< 0.01, ns, not significant.

Given that certain bacteria can exploit host mitophagy to enhance their intracellular survival, we next examined whether *P. gingivalis* initiates mitophagy to support intracellular survival in endothelial cells. At 24 h post-infection, our results showed that treatment with the mitophagy inhibitor Mdivi-1 suppressed, while the mitophagy activator CCCP increased the intracellular titer of *P. gingivalis* in HAECs (**Figure 5A**). Furthermore, we analyzed the number of *P. gingivalis* in HAECs using a confocal laser scanning microscope (CLSM). The results showed that CCCP treatment enhanced, while Mdivi-1 treatment decreased, the number of SYTO9-stained *P. gingivalis* within cells. Besides, there was no significant difference in the number or intracellular titer of *P. gingivalis* at 2 h post-infection across the treatment groups, suggesting that neither CCCP nor Mdivi-1 affected bacterial invasion (**Figures 5B, C**). Moreover, HAECs were transfected with either *Pink1* siRNA (20 nM) and negative control siRNA (20 nM) for 48 h, followed by *P. gingivalis* infection. We observed that PINK1 knockdown significantly reduced the intracellular survival of *P. gingivalis* (**Supplementary Figures 4A–C**; **Figure 5D**). These results indicated that *P. gingivalis*-initiated mitophagy promotes its intracellular survival.

To explore the role of lysosomal function in *P. gingivalis* survival, we further treated HAECs with BafA1 and the lysosomal agonist ML-SA1. ML-SA1 treatment suppressed while BafA1 treatment enhanced the intracellular titter and the number of SYTO9-stained *P. gingivalis* (**Figures 5E–G**). The results suggested that *P. gingivalis* impairs lysosomal function to prevent its xenophagic degradation, thereby promoting its intracellular survival.

### 3.5 *P. gingivalis* initiated mitophagy to suppress xenophagy in endothelial cells

**Figure 6:**
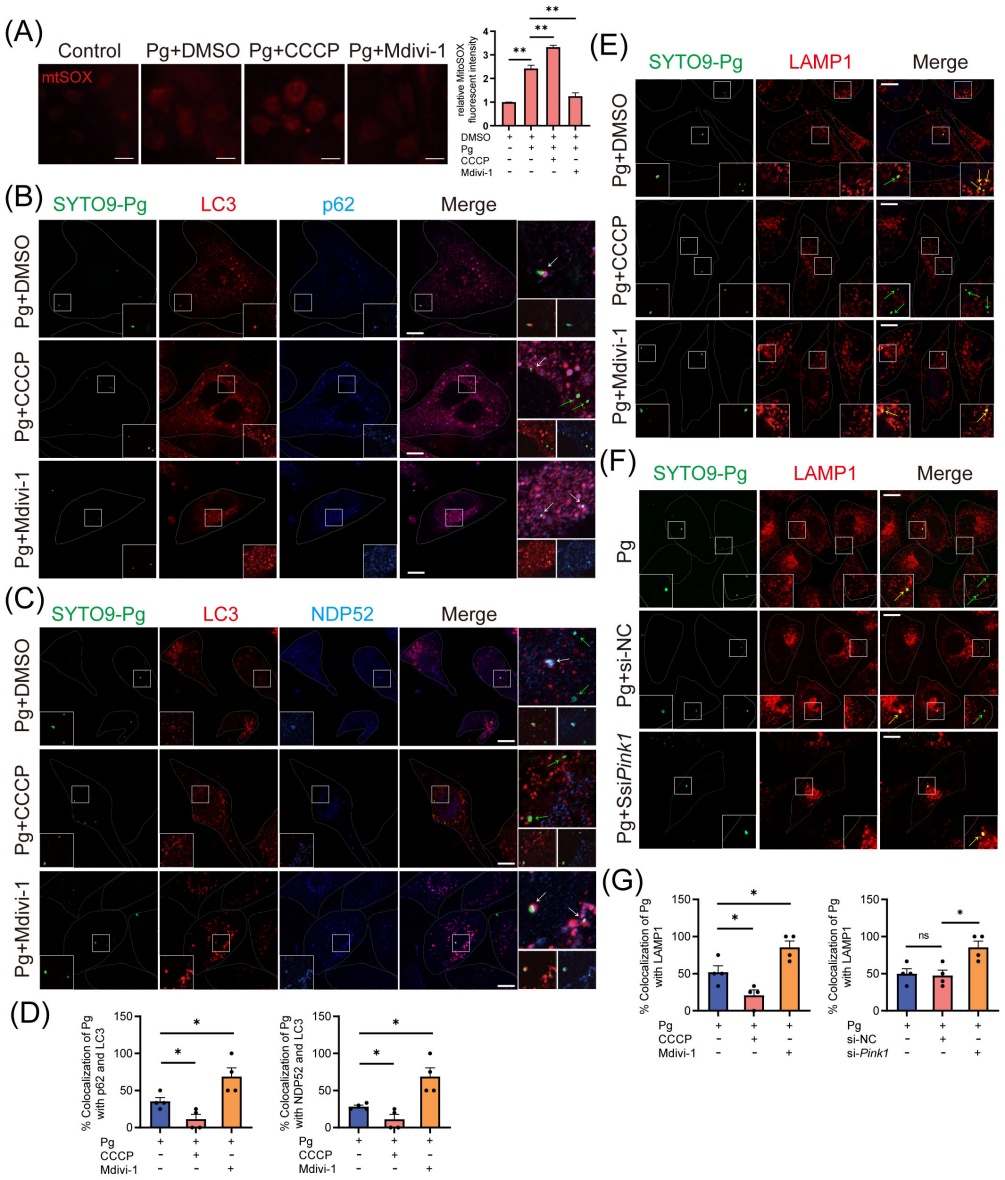
The initiation of mitophagy inhibits the host xenophagy, which targets *Porphyromonas gingivalis* to degrade. HAECs were treated with DMSO, CCCP (activation of mitophagy), or Mdivi-1 (inhibition of mitophagy), then infected with Pg (MOI = 100) for 24 h. **(A)** Representative fluorescence images and quantification of mitoSOX staining revealed the mitochondrial ROS production in each group. Scale bar: 65 μm. **(B, C)** Representative images and **(D)** quantification of LC3 (red)- Pg (green)- p62 (blue), LC3 (red)- Pg (green)- NPD52 (blue), and LAMP1 (red)- Pg (green) colocalization in DMSO, CCCP, or Mdivi-1 pretreated groups. Scale bar: 10 μm. **(E)** Representative images of SYTO9-stained Pg and LAMP1 colocalization in DMSO, CCCP, or Mdivi-1 pretreated groups. Scale bar: 10 μm. **(F)** HAECs were transfected with *Pink1* siRNA (20 nM) or negative control siRNA (20 nM) for 48 h and then infected with Pg (MOI = 100). Representative images of SYTO9-stained Pg and LAMP1 colocalization. Scale bar: 10 μm. **(G)** The quantification of Pg and LAMP1 colocalization. Data are presented as the mean ± SEM. *P < 0.05, and **P< 0.01, ns, not significant.

Subsequently, we sought to understand the mechanisms by which mitophagy supports *P. gingivalis* survival. Mitophagy has been recognized to regulate bacterial intracellular survival by modulating mtROS production or influencing xenophagy (Zhang et al., 2019; Song et al., 2022). We observed that *P. gingivalis* infection increased the level of mtROS in an MOI- and time-dependent manner (**Supplementary Figures 5A, B**), with CCCP treatment further exacerbating the mtROS levels in HAECs (**Figure 6A**). The results suggested that *P. gingivalis* initiated mitophagy to promote its survival in HAECs, independently of the pathway involving mtROS generation.

Finally, we investigated how mitophagy initiation affects xenophagy during *P. gingivalis* infection. Our study examined LC3 (red fluorescence)- *P. gingivalis* (green fluorescence) and p62/NDP52 (blue fluorescence)- *P. gingivalis* colocalization to evaluate autophagosome formation engulfing *P. gingivalis* and cargo receptors’ involvement. CLSM analysis showed that Mdivi-1 enhanced, while CCCP inhibited, the colocalization between LC3 and p62/NDP52-localized *P. gingivalis* in HAECs (**Figures 6B–D, F**), suggesting that the recruitment of p62 or NDP52 to *P. gingivalis* and xenophagosome formation were inhibited due to the initiation of mitophagy. Next, CLSM analysis revealed that the colocalization of LAMP1 with *P. gingivalis* could be promoted by Mdivi-1 and suppressed by CCCP (**Figures 6E, G**), indicating that mitophagy initiation inhibited the xenophagic degradation of *P. gingivalis*. To further strengthen this result, we observed the colocalization of LAMP1 with *P. gingivalis* in HAECs transfected with *Pink1* siRNA. The observation showed that PINK1 knockdown increased the colocalization of LAMP1 and *P. gingivalis* (**Figures 6F, G**). These results suggested that *P. gingivalis* initiates PINK1-mediated mitophagy to inhibit the formation of xenophagosomes, thereby enhancing its intracellular survival.

## 4 Discussion

**Figure 7:**
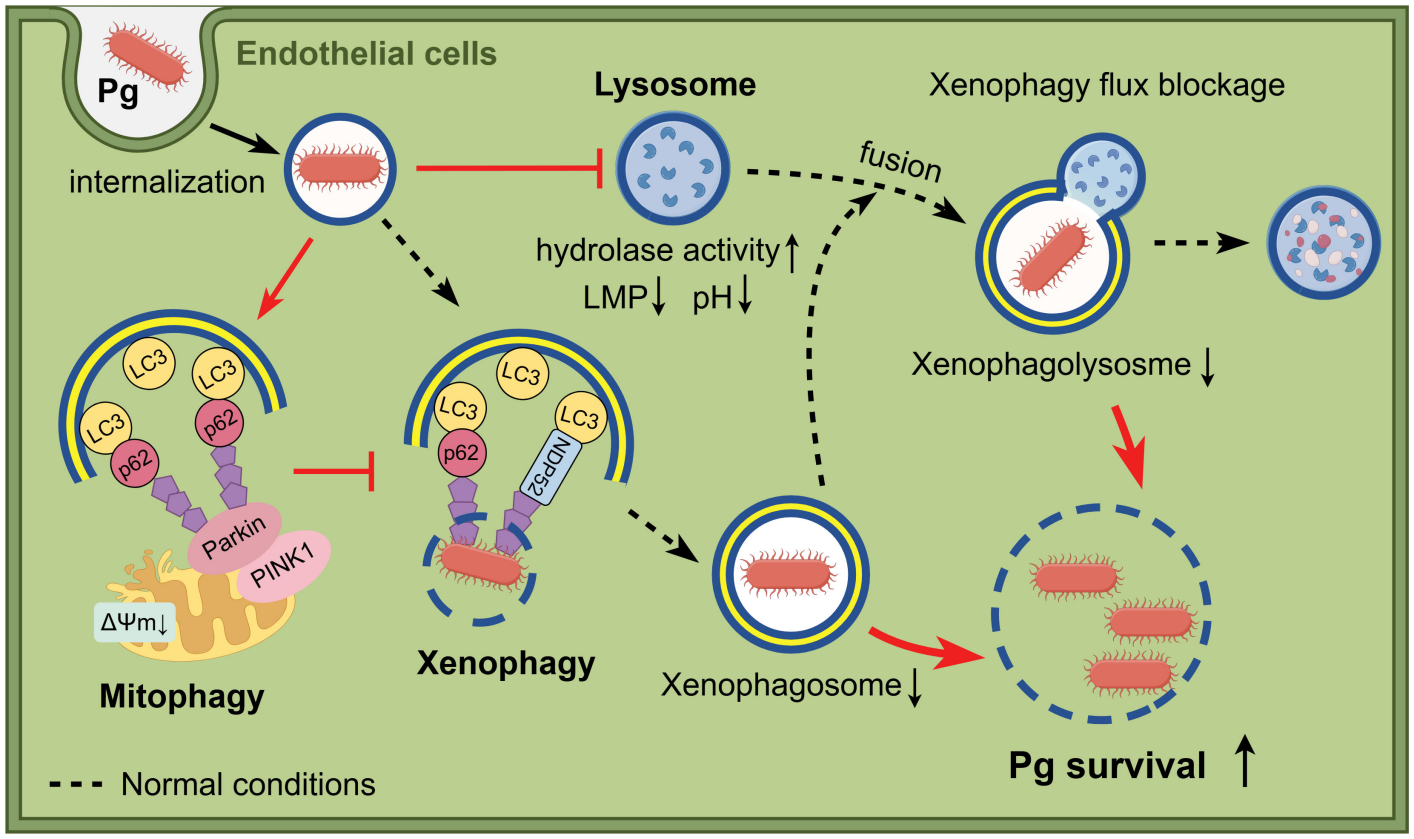
Schematic illustration of the study. When bacteria invade endothelial cells, *P. gingivalis* inhibits the formation of bacteria-containing xenophagosomes by initiating PINK1-Parkin-mediated mitophagy and simultaneously suppresses xenophagosome-lysosome fusion through lysosomal dysfunction, which results in enhanced intracellular survival. Figure created with cartoon components by Figdraw [www.figdraw.com].

Direct infection of *P. gingivalis* on endothelial cells has been linked to atherosclerosis. In this study, we found that *P. gingivalis* inhibited the formation of bacteria-containing xenophagosomes by initiating PINK1-Parkin-mediated mitophagy while simultaneously suppressing xenophagosome-lysosome fusion through lysosomal dysfunction. These findings contribute to our understanding of how *P. gingivalis* hijacks host mitophagy and lysosome function, shedding light on its role in pathogenesis and intracellular survival (**Figure 7**).

During bacterial invasion, autophagy serves as an innate immune response to restrict bacterial growth, while bacteria have evolved strategies to combat autophagy (Huang and Brumell, 2014; Sudhakar et al., 2019). Upon internalization, *P. gingivalis* initially localizes within a phagosome in host cells (Yamatake et al., 2007). Some *P. gingivalis* then damage the phagosome membrane, exposing themselves to the host cytosol, where they are targeted for degradation by xenophagy (selective autophagy) (Mostowy, 2013; Lee et al., 2018). However, others evade degradation by trafficking to replicative vacuoles through non-selective autophagy, thereby promoting intracellular survival (Dorn et al., 2001; Bélanger et al., 2006). Additionally, *P. gingivalis* may manipulate host-selective autophagy pathways to support survival (Shiheido-Watanabe et al., 2023).

Mitophagy, a selective form of autophagy that eliminates damaged mitochondria, can also be exploited by intracellular pathogens to evade host defenses (Zhang et al., 2019; Song et al., 2022). However, whether *P. gingivalis* exploits mitophagy to survive within endothelial cells remains unclear. Previous studies have shown that *P. gingivalis* inhibits mitophagy in oral squamous cell carcinoma cells and bone marrow-derived macrophages (Jiang et al., 2023; Sheridan et al., 2024). In contrast, our findings indicated that *P. gingivalis* initiates mitophagy at an early stage, leading to increased mitophagosome formation. However, it inhibits mitophagosome-lysosome fusion and the final degradation stage, likely due to lysosomal dysfunction in endothelial cells. Interestingly, we found that the differential effects of *P. gingivalis* on early and late stages of mitophagy/autophagy contribute to its survival within endothelial cells through distinct mechanisms. Consistent with the role of mitophagy in promoting bacterial intracellular survival in previous studies, we confirmed that *P. gingivalis* initiates mitophagy to enhance its survival in endothelial cells, although it only triggers the early stage of the mitophagy process.

Mitophagy is well-documented for promoting bacterial survival by reducing mtROS production (Zhang et al., 2019; Gao et al., 2024; Nan et al., 2024). In our study, while *P. gingivalis* initiated mitophagy in HAECs, the mitophagy flux was ultimately blocked. As a result, damaged mitochondria accumulated, leading to increased mtROS levels, which couldn’t be reversed by the mitophagy activator. This suggested that *P. gingivalis* may not promote its survival by regulating mtROS levels in endothelial cells. Other bacteria can manipulate mitophagy to favor persistent infection through another strategy. Xenophagy, a form of selective autophagy closely linked to bacterial survival, shares several key molecules with mitophagy, including autophagy receptors like p62 and NDP52, and the E3 ubiquitin ligases like Parkin. This overlap implies potential crosstalk between mitophagy and xenophagy (Rubio-Tomás et al., 2023). A recent study demonstrated that *M. bovis* induces mitophagy to suppress host xenophagy, thereby facilitating its persistence (Vargas et al., 2023) Likewise, our findings revealed that *P. gingivalis* inhibited xenophagy by initiating mitophagy in HAECs. When mitophagy was inhibited using Mdivi-1, we observed increased recruitment of p62 and NDP52 to *P. gingivalis*, as indicated by enhanced colocalization. These receptors were directly bound to LC3, enabling the delivery of *P. gingivalis* to xenophagosomes and promoting its clearance when mitophagy was suppressed.

Lysosomes play a crucial role in the fusion and degradation of both mitophagosomes and xenophagosomes, which determine whether damaged mitochondrions are cleared and whether bacteria survive (Miao et al., 2015; Hu et al., 2019; Naskar et al., 2023). A study has shown that *P. gingivalis* blocks autophagosome-lysosome fusion by cleaving VAMP8 (a protein located on lysosomes) through its gingipains and by promoting lysosomal efflux (Shiheido-Watanabe et al., 2023; Liu et al., 2023). In contrast, we assessed lysosomal function and found that *P. gingivalis* induced lysosomal dysfunction. This dysfunction was characterized by increased lysosomal number, lysosomal membrane permeabilization, disruption of the lysosomal acidic environment, and reduced enzymatic activity (CTSB, CTSD, and ACP). Interestingly, the increased lysosomal number may reflect a compensatory response to these lysosomal function defects (Zhang et al., 2021). Additionally, we confirmed that activating lysosome function reduced the survival of *P. gingivalis* in HAECs, while inhibition of lysosomal function had the opposite effect, emphasizing lysosomal dysfunction as a potential mechanism by which *P. gingivalis* promotes its intracellular survival in endothelial cells.

Our study demonstrated that *P. gingivalis* initiates mitophagy and impairs lysosomal function in HAECs, inhibiting xenophagosome formation and its fusion with lysosomes. Both processes contribute to the intracellular survival of the bacterium. However, several limitations of our study should be noted. The specific virulence factors and mechanisms by which *P. gingivalis* initiates mitophagy to suppress xenophagy are yet to be identified, and the relevance of these mechanisms *in vivo* is unclear. Additionally, our analysis focused on a single strain of *P. gingivalis*, necessitating further studies to investigate strain-specific variations.

In conclusion, this study enhances our understanding of how *P. gingivalis* interacts with endothelial cells, particularly its manipulation of mitophagy and lysosomal dysfunction to promote survival. Our findings provide new insights for understanding the intracellular survival mechanism of *P. gingivalis* in endothelial cells.

## Data Availability

The original contributions presented in the study are included in the article/**Supplementary Material**. Further inquiries can be directed to the corresponding authors.
