# Supplementary material for: Porphyromonas gingivalis hijacks mitophagy and lysosomal function to persist in endothelial cells
Source: Front Cell Infect Microbiol. 2025 Aug 8;15:1613366. doi: 10.3389/fcimb.2025.1613366 (PMC12370697; doi:10.3389/fcimb.2025.1613366)
Supplement: Supplementary file 1 [file DataSheet1.docx]

Supplementary Material

# Appendix

## Materials and Methods

## Mitochondrial DNA Copy Number Analysis

Genomic DNA was extracted from HAECs using the Universal Genomic DNA Extraction Kit (CINOTOHI, Changsha, China) according to the instructions of the manufacturer. DNA was amplified by real-time quantitative PCR using the HiScript II Q RT SuperMix (Vazyme, China). The mitochondrial DNA (mtDNA) copy number was measured as the mtDNA:nDNA ratio. The mtDNA level was reflected by the *mt-Atp6* gene with the forward primer 5’-AGGCACACCTACACCCCTTA-3’ and the reverse primer 5’-GATATTGCTAGGGTGGCGCT-3’, while nDNA level was reflected by the *Rpl13a* gene with the forward primer 5’-GCCATCGTGGCTAAACAGGTA-3’ and the reverse primer 5’-GTTGGTGTTCATCCGCTTGC-3’. The results were obtained by the 2^−△△CT^ method.

## Analysis of Mitochondrial Membrane Potential (MMP)

The effect of *P. gingivalis* on the MMP of HAECs was detected using a mitochondrial membrane potential assay kit with JC-1 (Beyotime, Shanghai, China) according to the instructions of the manufacturer. The HAECs were observed under a fluorescence microscope. Each experiment was performed in triplicate.

# Supplementary Figures and Tables

## Supplementary Tables

**Supplementary Table 1**

Primary antibody information for Western Blotting

| Antibody | Vendor | Catalog/Institution | Concentration |
| --- | --- | --- | --- |
| mouse anti-LAMP1 | Santa Cruz | sc-20011 | 1:1000 |
| mouse anti-Parkin | Cell Signaling Technology | 4211 | 1:1000 |
| rabbit anti-PINK1 | Cell Signaling Technology | 6946 | 1:1000 |
| mouse anti-LC3B | Cell Signaling Technology | 83506 | 1:1000 |
| rabbit anti-LC3B | Cell Signaling Technology | 2775 | 1:1000 |
| rabbit anti-p62 | proteintech | 18420-1-AP | 1:5000 |
| rabbit anti-TOM20 | proteintech | 11802-1-AP | 1:5000 |
| rabbit anti-COX IV | proteintech | 11242-1-AP | 1:5000 |
| rabbit anti-CTSD | proteintech | 21327-1-AP | 1:5000 |
| rabbit anti-CTSB | proteintech | 12216-1-AP | 1:1000 |

**Supplementary Table 2**

Primary antibody information for IF

| Antibody | Vendor | Catalog/Institution | Concentration |
| --- | --- | --- | --- |
| mouse anti-LAMP1 | Santa Cruz | sc-20011 | 1:250 |
| mouse anti-Parkin | Cell Signaling Technology | 4211 | 1:200 |
| rabbit anti-PINK1 | Cell Signaling Technology | 6946 | 1:200 |
| mouse anti-LC3B | Cell Signaling Technology | 83506 | 1:250 |
| rabbit anti-LC3B | Cell Signaling Technology | 2775 | 1:250 |
| rabbit anti-p62 | proteintech | 18420-1-AP | 1:500 |
| rabbit anti-TOM20 | proteintech | 11802-1-AP | 1:250 |
| rabbit anti-Tim23 | proteintech | 11242-1-AP | 1:500 |
| rabbit anti-NDP52 | Abcam | ab68588 | 1:200 |

## Supplementary Figures


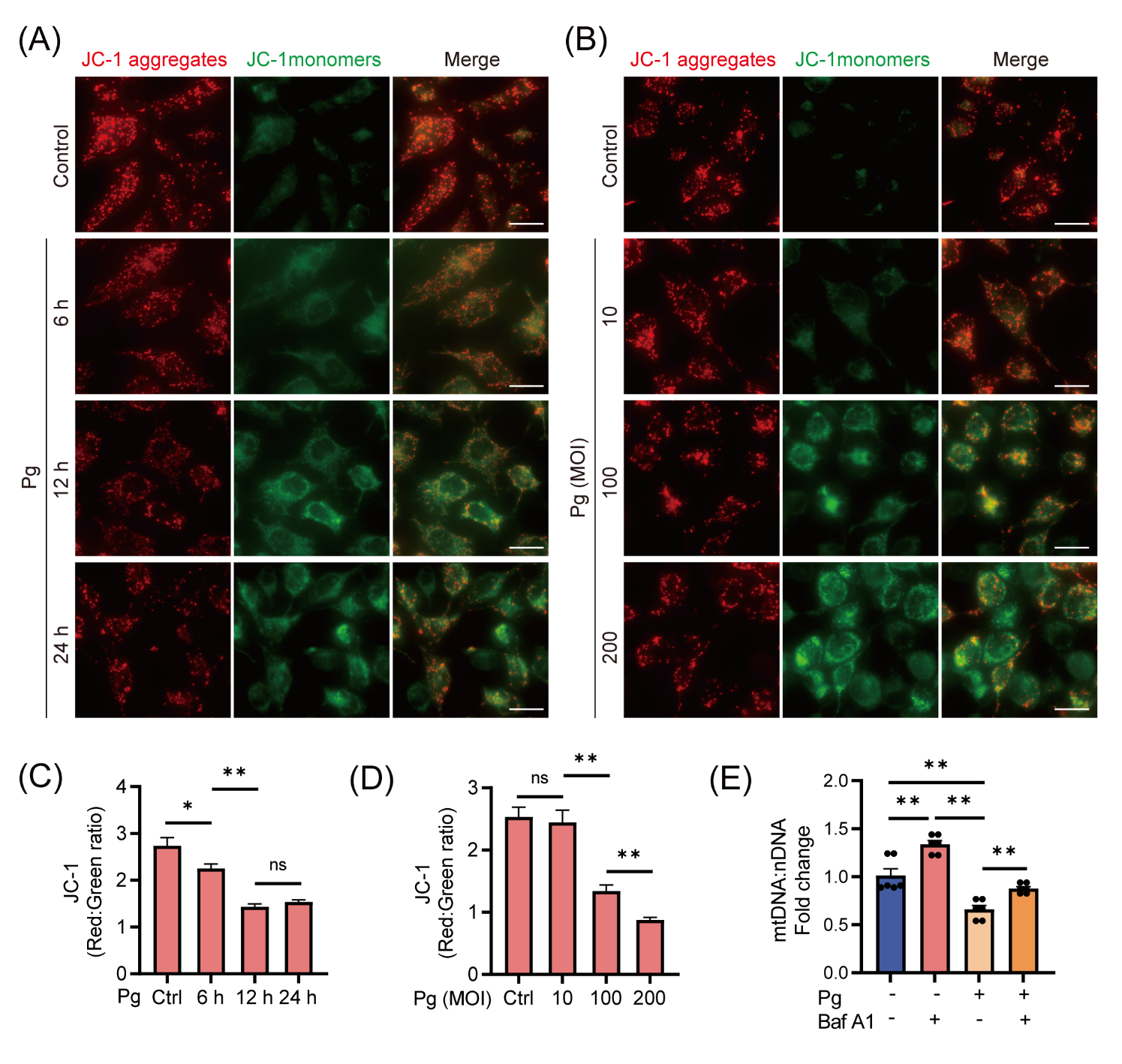


**Supplementary Figure 1.** Effects of *P. gingivalis* on MMP level and mitochondrial DNA (mtDNA) copy number. (A, B) HAECs were infected with *P. gingivalis* in a time- and MOI-dependent manner. Representative fluorescence images and (C, D) quantification of JC-1 staining revealed the MMP in each group. Scale bar: 20 μm. (E) The relative level of mtDNA significantly decreased in HAECs exposed to Pg, as measured by mtDNA/nDNA analysis (n=6). Data are presented as the mean ± SEM. **P* < 0.05, and ***P*< 0.01, ns, not significant.


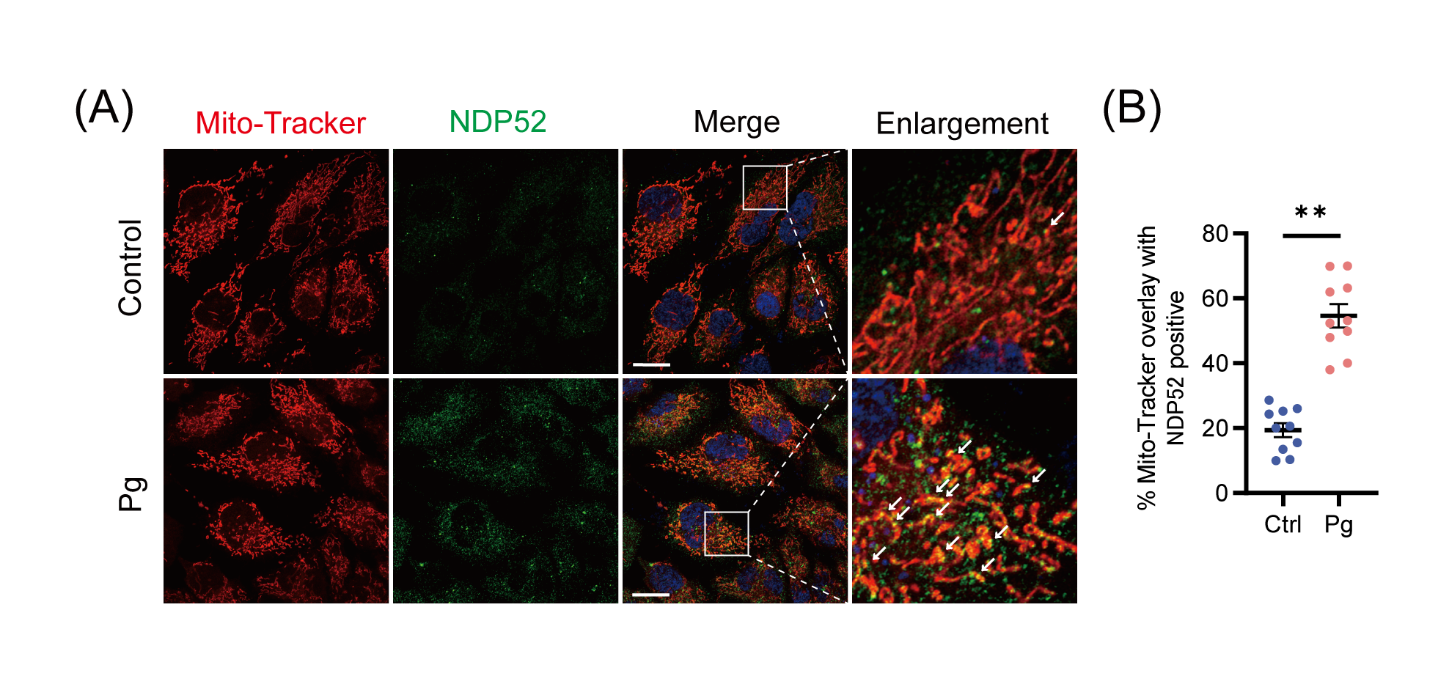


**Supplementary Figure 2.** Mitophagosome formation accumulated upon *Porphyromonas gingivalis* infection. HAECs were infected with Pg at an MOI of 100 for 24 h. (A) Representative images and (B) quantification of Mito-Tracker (red) and NDP52 (green) colocalization by CLSM. Scale bar: 15 μm. (n = 10-15 cells per group). Data are presented as the mean ± SEM. **P* < 0.05, and ***P*< 0.01, ns, not significant.


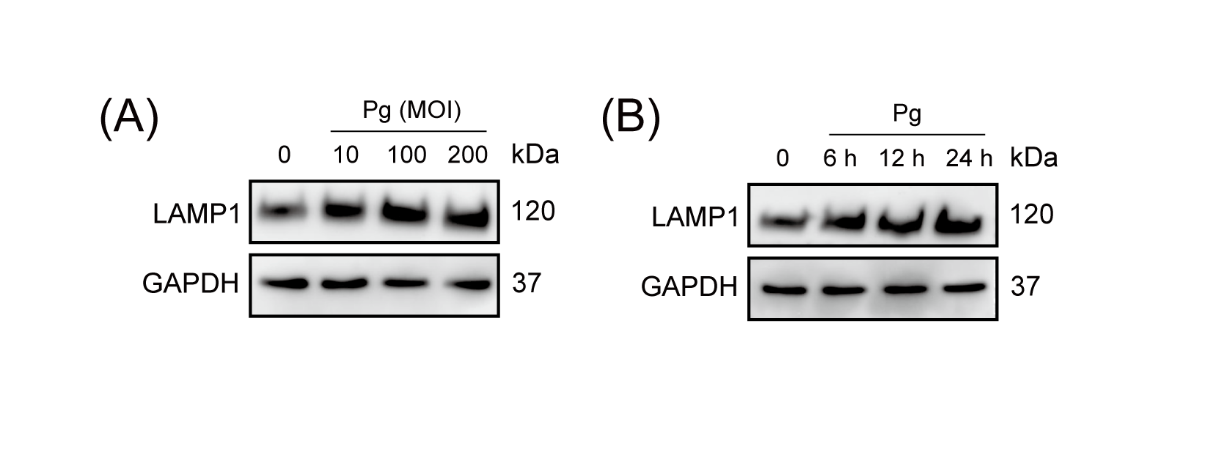


**Supplementary Figure 3.** Effects of *P. gingivalis* on MMP level and mitochondrial DNA (mtDNA) copy number. (A, B) HAECs were infected with *P. gingivalis* in a time- and MOI-dependent manner. Representative western blot and (C) quantification of LAMP1 protein expression in each group.


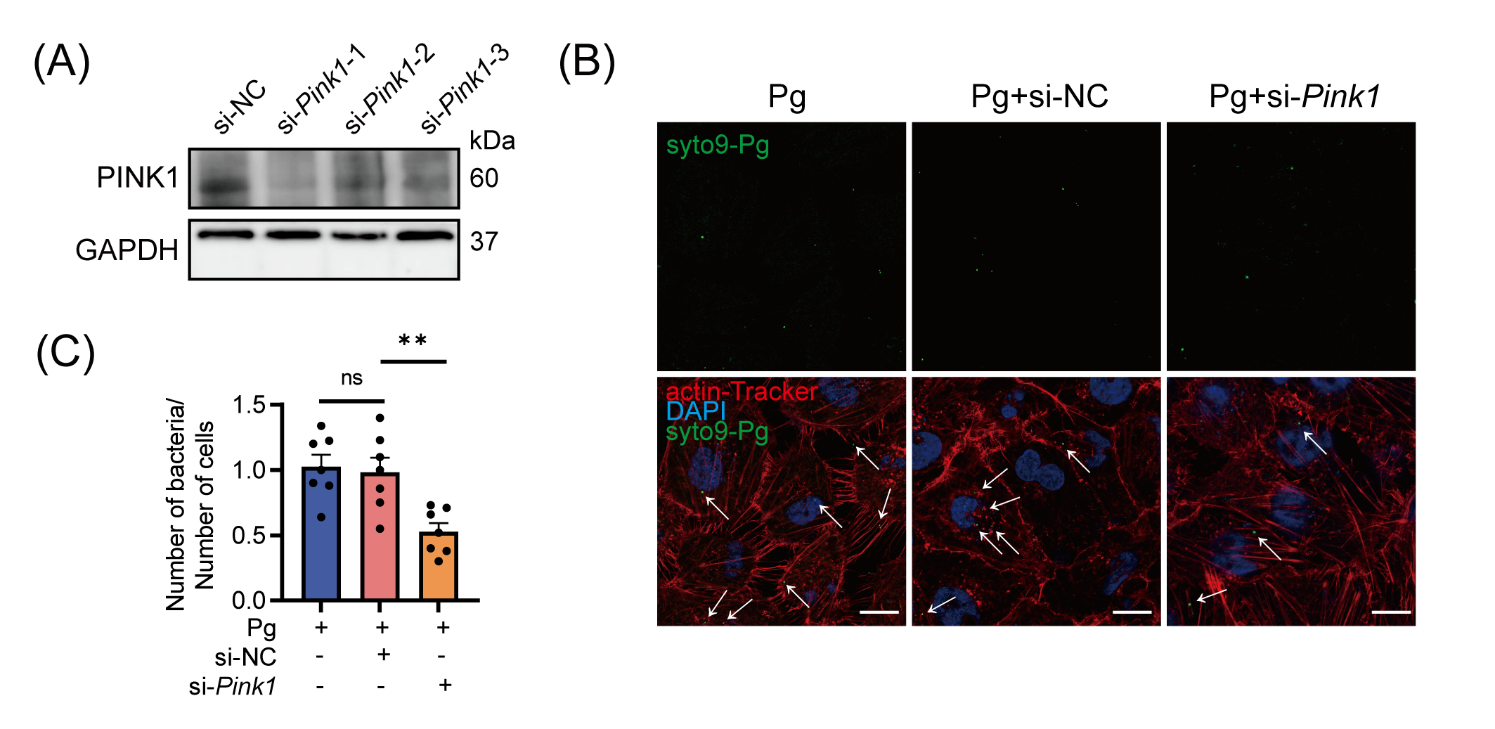


**Supplementary Figure 4.** The gene silence of PINK1 decreased the intracellular survival of *Porphyromonas gingivalis* in endothelial cells. HAECs were transfected with *Pink1* siRNA-1/2/3 (20 nM) or negative control siRNA (20 nM) for 48 h. (A) The efficacy of the gene knockout was validated by Western blot, and we selected the si-*Pink1*-1 plasmid for subsequent experiments. (B) Representative fluorescence images and (C) quantification of intracellular SYTO9-stained Pg load by CLSM. Scale bar: 15 μm. Cells in at least 6 fields were counted in each group. Data are presented as the mean ± SEM. **P* < 0.05, and ***P*< 0.01, ns, not significant.


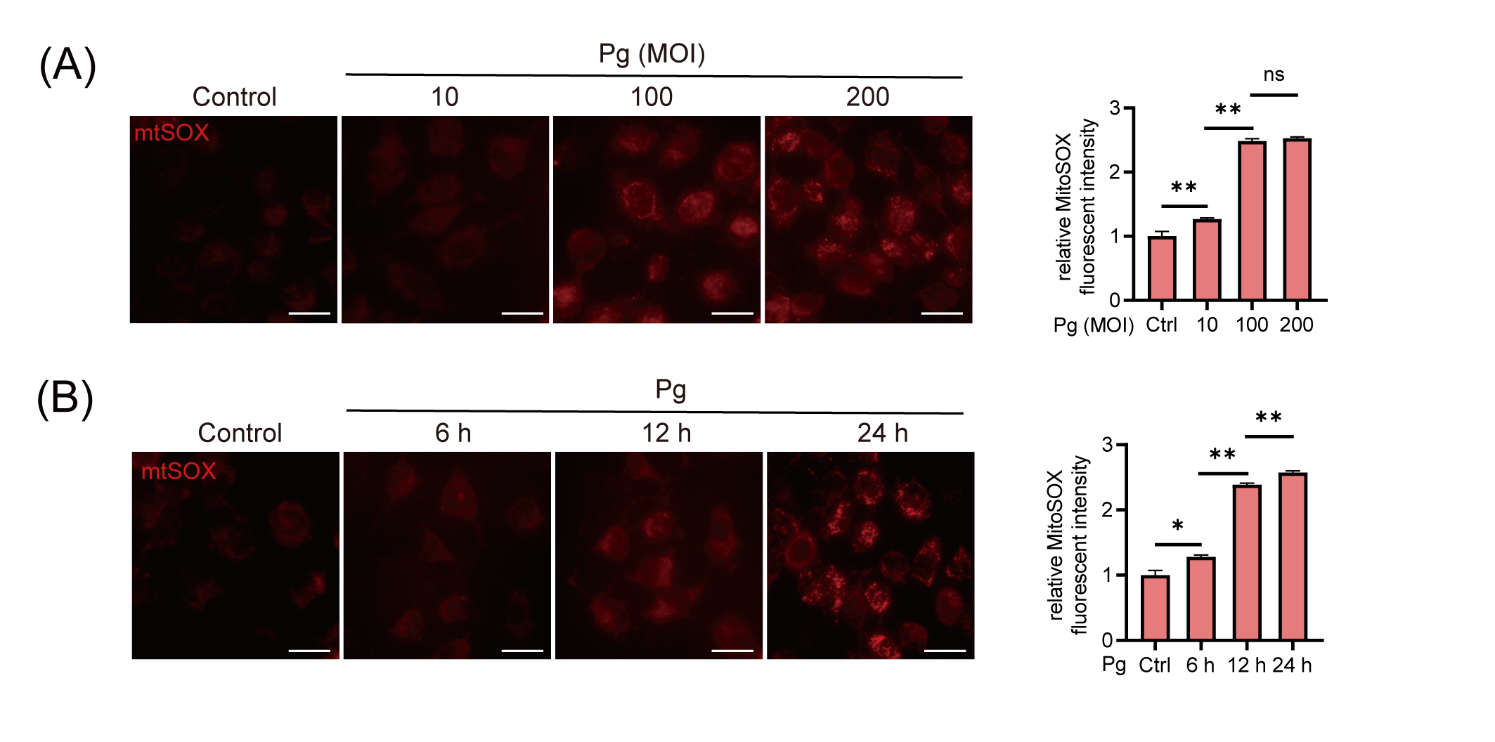


**Supplementary Figure 5.** Effects of *P. gingivalis* on mtROS level. (A, B) HAECs were infected with *P. gingivalis* in a time- and MOI-dependent manner. Representative fluorescence images and (C, D) quantification of mitoSOX staining revealed the mtROS production in each group. Scale bar: 65 μm. Data are presented as the mean ± SEM. **P* < 0.05, and ***P*< 0.01, ns, not significant.
